# Targeted endoscopic ultrasound-guided vascular angiotherapy for refractory upper gastrointestinal bleeding due to an aberrant collateral vessel

**DOI:** 10.1055/a-2830-4168

**Published:** 2026-03-25

**Authors:** Amine Achemlal, Shrikant Mukewar, Bhushan Bhaware, Shubhankar Godbole, Saurabh Mukewar

**Affiliations:** 1 Division of Gastroenterology and Digestive Endoscopy, Midas Hospital, Nagpur, India; 2 479569 Gastroenterology Unit I, Mohammed V Military Instruction Hospital, Rabat, Morocco

A 21-year-old man with no significant past medical history presented with a 5-month history of recurrent hematemesis, including several episodes of massive upper gastrointestinal bleeding. He required transfusion of 10 units of packed red blood cells and underwent three upper gastrointestinal endoscopies during this period. All examinations demonstrated a persistent gastric fundal protuberance with inflammatory changes on biopsies. The lesion was suspected to be a gastrointestinal stromal tumor and later misinterpreted as an isolated gastric varix, leading to cyanoacrylate injection without clinical benefits. An externally performed endoscopic ultrasound (EUS) excluded both diagnoses but failed to identify the source of bleeding.

**Fig. 1:**
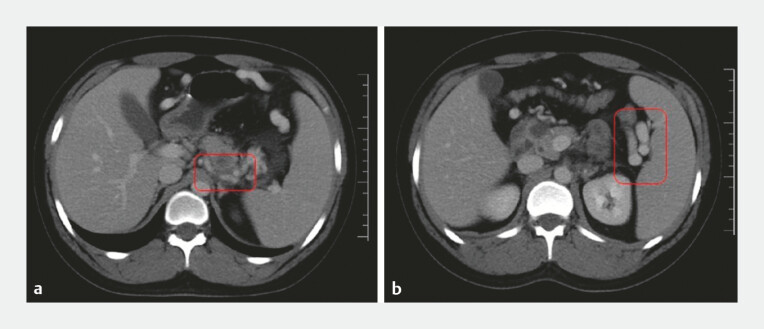
Contrast-enhanced computed tomography showing **a** focal narrowing of the splenic vein with partial thrombosis and **b** associated splenomegaly with prominent, dilated collateral vessels at the splenic hilum.

The patient was subsequently referred to our center for further evaluation. A computed tomographic scan revealed segmental narrowing with partial thrombosis of the splenic vein, marked dilatation of collateral vessels at the splenic hilum, and splenomegaly, without any active bleeding ( Fig. 1 ).

**Fig. 2:**
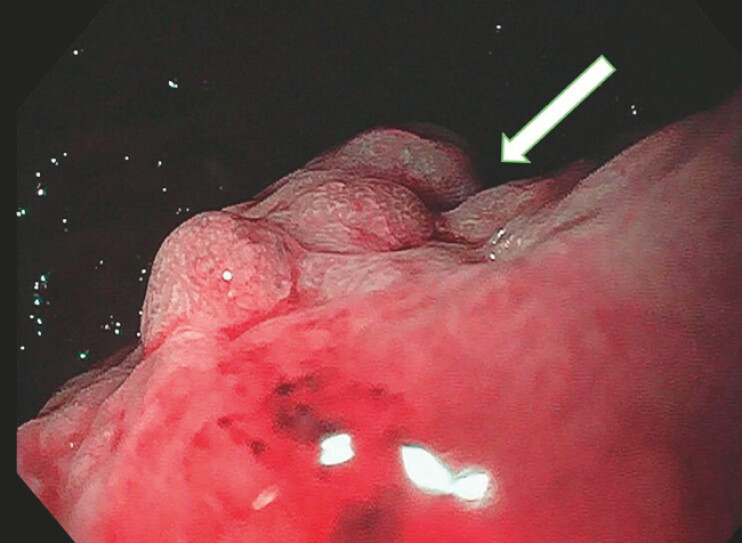
Upper gastrointestinal endoscopy showing a mucosal protuberance (arrow) in the proximal gastric body along the greater curvature, without suspicious features on narrow-band imaging.

Upper gastrointestinal endoscopy demonstrated a mucosal protuberance in the proximal gastric body along the greater curvature, with normal surrounding mucosa ( Fig. 2 ).

**Fig. 3:**
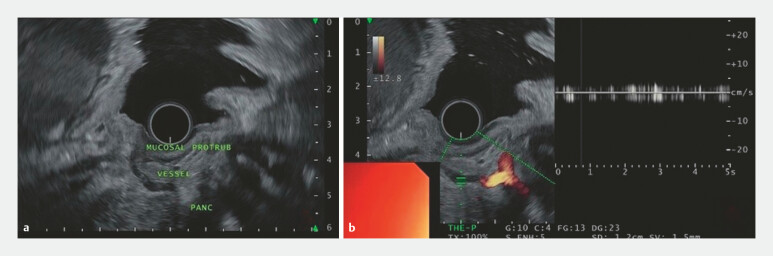
**a** Radial EUS showing the vessel traversing the gastric muscularis propria toward the lesion. **b** Power Doppler imaging demonstrated active blood flow.

Radial EUS identified an isoechoic lesion measuring 15 × 13 mm, predominantly confined to the mucosal layer. A linear anechoic structure traversing the muscularis propria toward the lesion was visualized ( Fig. 3 ). Power Doppler imaging confirmed the active blood flow within this vessel, measuring approximately 1.2 mm in diameter. These findings were consistent with an aberrant collateral vessel arising from splenic vein thrombosis.

Given the high likelihood that this vessel was responsible for the recurrent bleeding, EUS-guided vascular therapy was performed (using a 19G EUS FNA needle). One cc of cyanoacrylate glue was successfully injected into the vessel under EUS guidance, resulting in immediate obliteration and confirmed the absence of Doppler flow ( Video 1 ). The patient experienced no further episodes of gastrointestinal bleeding, and hemoglobin levels remained stable during 3 months of follow-up. This case highlights EUS-guided angiotherapy 1 2 as a rescue therapy where no other management options exist.

Targeted endoscopic ultrasound-guided vascular angiotherapy for an aberrant collateral vessel.Video 1

Endoscopy_UCTN_Code_CCL_1AB_2AD_3AZ Endoscopy_UCTN_Code_TTT_1AS_2AL
